# Solvent-free phase-vanishing reactions with PTFE (Teflon^®^) as a phase screen

**DOI:** 10.3762/bjoc.5.75

**Published:** 2009-12-09

**Authors:** Kevin Pels, Veljko Dragojlovic

**Affiliations:** 1 Wilkes Honors College of Florida Atlantic University, 5353 Parkside Drive, Jupiter, FL 33458, USA

**Keywords:** bromination, phase-vanishing, PTFE, solvent-free, stilbene

## Abstract

In a solvent-free phase-vanishing reaction with PTFE (polytetrafluoroethylene, Teflon^®^) tape as the phase screen, a thermometer adapter is utilized to insert a PTFE-sealed tube into the vapor phase above the substrate. Besides avoiding use of solvents, the experimental design is not dependent upon the densities of the reactants and the procedure generates little or no waste while providing the reaction products in high yield and in high purity.

## Findings

Phase-vanishing (PV) reactions are triphasic reactions, which involve a reagent, a liquid perfluoroalkane and a substrate [1–16]. In a test tube, a liquid perfluoroalkane acts as a phase screen to separate the two reactants, one of which is more dense than the screen and the other one that is less dense. If both reactants are less dense than the phase screen, one can carry out the reaction in a U-tube and if both reactants are denser than the screen, the reaction can be carried out in an inverted U-tube. As the reagent diffuses through the perfluoroalkane, it reaches the substrate and reacts with it. In the course of the reaction, the reagent is used up (“vanishes”). One disadvantage of PV reactions is that a commonly used phase screen, fluorinert liquid FC-72 (C_6_F_14_), has a high global warming potential (GWP ~10,000) and atmospheric lifetime of 3200 years [17]. In addition, some solvents, reagents and reaction products are partially soluble in FC-72 [13–16,18], which limits our ability to either reuse or recycle it.

Ryu and Curran have reported benefits in using heavier liquid perfluoro compounds as phase screens [19]. Gladysz used Teflon^®^ tape to deliver and recover a fluorous catalyst [20]. Recently, we reported preliminary results on use of a solid perfluoro compound, PTFE (polytetrafluoroethylene, Teflon^®^) tape, as a phase screen between the two liquid phases [21]. PTFE offers some advantages compared to a liquid perfluoro phase screen: PTFE tape is inexpensive, easy to use and may be reused. In addition, experimental design is not dependent upon densities of the reactants. However, in some cases, such as when iodine monochloride was used as a reagent or when insoluble products formed an impermeable barrier on the PTFE tape, this method was not suitable. Finally, PV-PTFE reaction design required use of a solvent. To address the described shortcomings and to make the design more environmentally friendly, we have developed solvent-free PV-PTFE reactions.

**Figure 1:**
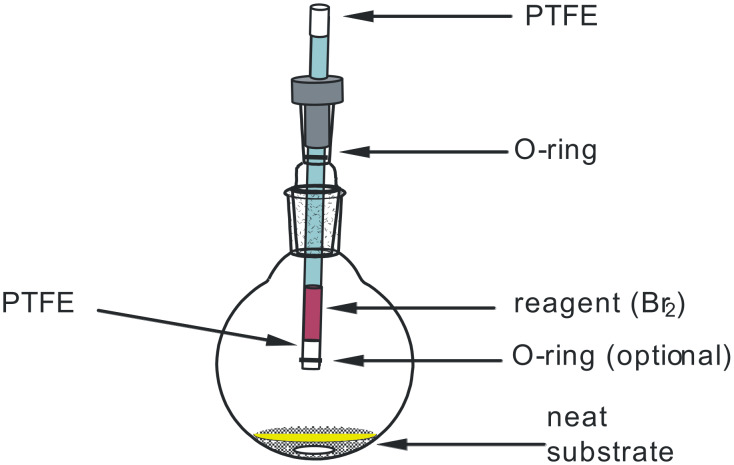
Solvent-free PV-PTFE reaction apparatus.

An apparatus used to carry out solvent-free PV-PTFE reactions is shown in Figure 1. Neat substrate was placed in the flask and a PTFE-sealed tube filled with the reagent was placed into the vapor phase above it. A thermometer adapter, with a reagent delivery tube in place of thermometer, was used for this purpose. A loaded tube inserted into an empty closed flask could be stored for a future use for several days as long as there were no leaks. There was equilibrium between the liquid reagent in the tube and its vapors in the flask. The reagent diffused through PTFE tape only if the vapors were consumed or removed (e.g. by being used up in a reaction, dissolved in a solvent or blown away with nitrogen). When a substrate, which consumes the reagent vapors, is placed in the reaction vessel, the reagent rapidly diffuses out of the tube as its vapors are consumed. With the reactions that evolve gases (HCl, HBr), one should provide an appropriate outlet and possibly a gas trap (additional details are provided in Supporting Information File 1). A gas outlet was useful even when there was no evolution of a gas as sometimes there was a drop in pressure as a volatile substrate reacted to give a non-volatile product.

**Figure 2:**
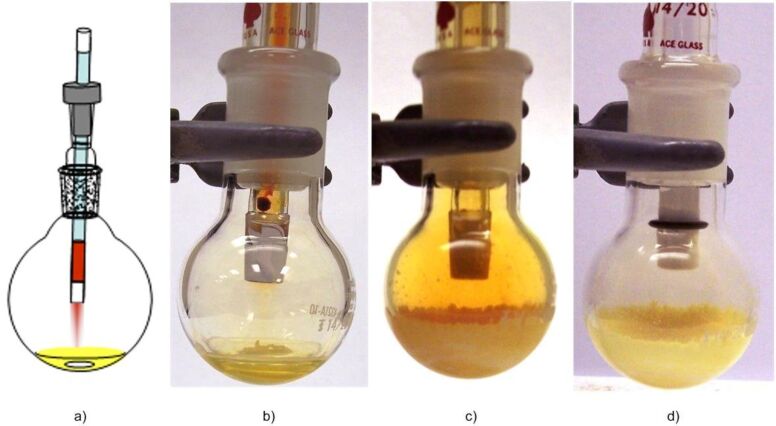
Bromination of *cis*-stilbene. a) scheme of the reaction apparatus, b) reaction mixture (note a thin stream of bromine vapors flowing straight down), c) bromination was completed when the color of bromine vapors persisted, d) after a work-up tube was inserted bromine vapors were consumed.

We chose bromination reactions as a model system for the initial studies of solvent-free PV-PTFE reaction as bromine vapors are visible and the progress of a reaction could be easily monitored (Table 1). In the cource of bromination of *cis*-stilbene, shown in Figure 2, initially bromine vapors were rapidly consumed (Figure 2a and Figure 2b). The end of the reaction was indicated by the presence of an excess of bromine vapors in the flask (Figure 2c). At that point, the bromine delivery tube was replaced with a reaction work-up tube (a PTFE-sealed tube filled with aqueous thiosulfate) (Figure 2d). Organic reactions on powdered solid substrates are known [22] and bromination of solid *trans*-stilbene under solvent-free PV-PTFE conditions worked well (Table 1, entry 6). Due to its lower reactivity, bromine vapors were present in the flask throughout the reaction and the reaction time was longer compared to bromination of liquid substrates (Table 1). The end point of a reaction was determined by removing the delivery tube and weighing the reaction flask.

**Table 1:** Addition reactions of bromine under solvent-free PV-PTFE conditions.

Entry	Substrate	Product (% yield)^a^	Time (min)

1	cyclohexane (**1**)	*trans*-1,2-dibromocyclohexane (**2**) (94)	10
2	1-octene (**3**)	1,2-dibromooctane (**4**) (95)	10
3	2,3-dimethyl-2-butene (**5**)	2,3-dibromo-2,3-dimethylbutane (**6**) (97)	5
4	3,3-dimethyl-1-butene (**7**)	1,2-dibromo-3,3-dimethylbutane (**8**) (94)	8
5	*cis*-stilbene (**9**)	dibromostilbene (**10**) (100^b^)	15
6	*trans*-stilbene (**11**)	dibromostilbene (**12**) (98, *erythro*/*threo* 96:4^c^)	60

^a^Isolated yields. ^b^Crude product was composed of 91% D,L-dibromostilbene, 5% *trans*-stilbene and 4% *cis*-stilbene (^1^H NMR analysis). ^c^Ratio determined by ^1^H NMR.

Cyclohexene and 1-octene gave the corresponding dibromo derivatives cleanly and in good yields (Table 1, entries 1 and 2). 2,3-Dimethyl-2-butene and 3,3-dimethyl-1-butene are highly volatile substrates and, although the reaction was fast, for clean products one had to conduct the reaction in the dark and, in the case of 2,3-dimethyl-2-butene, at a low temperature (Table 1, entries 3 and 4). As 2,3-dibromo-2,3-dimethylbutane is a solid, better results were obtained when the delivery tube was kept at a distance (~4 cm on a 2 mmol reaction scale) then close (~2 cm) to the substrate. If a tube was too close, it would get coated with a solid product, which dissolved bromine from the tube to give numerous byproducts. Combination of a low temperature (reduced volatility of the substrate) and greater distance resulted in formation of a pure product.

**Scheme 1:**
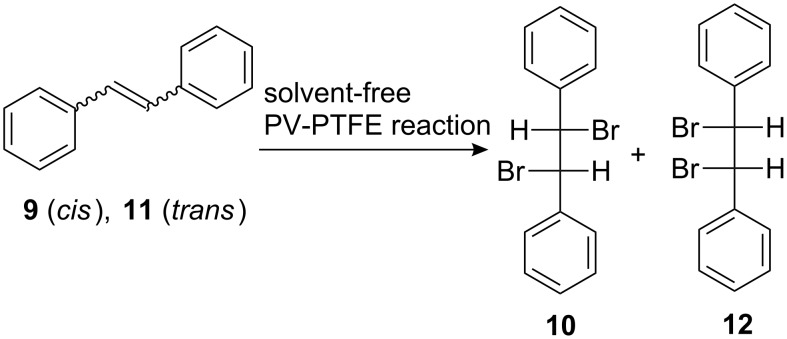
Bromination of stilbenes.

The stereochemistry of bromination of *cis*-stilbene (**9**) is more complex compared to *trans*-stilbene (**11**) (Scheme 1). While bromination of *trans*-stilbene is a stereoselective reaction, that usually is not the case with *cis*-stilbene and the reaction product is a mixture of D,L- and *meso*-dibromostilbenes [23–25]. Besides the common mechanism involving a bromonium ion as an intermediate, bromination of *cis*-stilbene may involve the corresponding carbocation [24–25] as well as isomerization, followed by a subsequent bromination of *trans*-stilbene. Other research groups have been able to improve selectivity of bromination by using tridecylmethylphosphonium tribromide [12] or pyridinium hydrobromide perbromide [23]. Still, a considerable amount (10–20%) of the *meso*-dibromostilbene (**12**) was produced. Bromination of *cis*-stilbene under solvent-free PV-PTFE conditions gave an almost pure D,L-dibromostilbene (**10**), which was accompanied by small amounts of *cis*- and *trans*-stilbenes (Table 1, entry 5). *trans*-stilbene (**11**) could not be brominated under PV-PTFE conditions in solution [21]. The reaction product (**12**) was insoluble and an impermeable coating of it on the PTFE tape prevented further reaction. A solvent-free vapor phase reaction gave the expected *meso*-dibromostilbene (**12**) in a good yield (Table 1, entry 6).

Depending on the reaction conditions, bromination of phenol gave 4-bromophenol, 2,4-dibromophenol or 2,4,6-tribromophenol. Reaction of phenol with 1.1 equiv of bromine yielded 4-bromophenol in a good yield. A similar result was obtained with an excess of bromine provided that the reaction time was kept short (Table 2, entries 1 and 2). Reaction with either 2.2 equiv, or in a closed system, resulted in formation of 2,4-dibromophenol (Table 2, entries 3 and 4). Reaction in a vessel open to air resulted in formation of about equal amount of 2,4-dibromo and 2,4,6-tribromophenols (Table 2, entry 5). Finally, reaction in the presence of a small amount of water resulted in formation of 2,4,6-tribromophenol (Table 2, entry 6).

**Table 2:** Bromination of phenol (**13**) under solvent-free PV-PTFE conditions.

Entry	Conditions	Product (% yield)^a^	Time (min)

1	Br_2_ (1.1 equiv)	4-bromophenol (**14**) (89)	10
2	Br_2_ (excess)	**14** (86)	10
3	Br_2_ (2.2 equiv)	2,4-dibromophenol (**15**) (82)	30
4	Br_2_ (excess), closed system	**15** (90)	30
5	Br_2_ (excess), open to air	(**15**) (41) + 2,4,6-tribromophenol (**16**) (46)	240
6	Br_2_ (excess), H_2_O	**16** (91)	60

^a^Isolated yields.

Bromination of 4-pentenoic acid (**17**) gave the corresponding lactone **18** as the major product accompanied by some dibromoacid **19** (Table 3, entry 1). Reaction of the same compound with iodine monochloride gave only the corresponding lactone in a very good yield (Table 3, entry 2). Bromination of 3-butenoic acid (**21**) gave the corresponding lactone **22** as the minor product while the dibromoacid **23** was the major product.

**Table 3:** Halolactonization and tandem Diels-Alder/Halolactonization reactions under solvent-free PV-PTFE conditions.

Entry	Substrate	Conditions	Product (% yield)^a^	Time (min)

1	4-pentenoic acid (**17**)	Br_2_ (1.1 equiv)	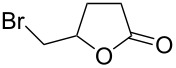 **18** (56) 4,5-dibromopentanoic acid (**19**) (28)	15
2	**17**	ICl (2.5 equiv)	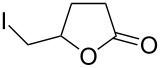 **20** (90)	60
3	3-butenoic acid (**21**)	Br_2_ (1.1 equiv)	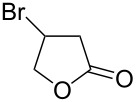 **22** (21) 3,4-dibromobutanoic acid (**23**) (67)	10
4	cyclopentadiene (**24**), acrylic acid (**25**)	ICl (1.2 equiv)	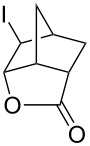 **26** (38)	60
5	cyclopentadiene (**24**), dimethyl fumarate (**27**)	ICl (1.2 equiv)	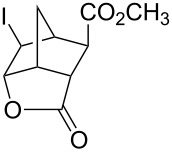 **28** (87)	360

^a^Isolated yields.

We examined the possibility of tandem reactions with a solvent-free Diels-Alder/halolactonization reaction. Cyclopentadiene (**24**) and acrylic acid (**25**) were combined in a flask with stirring and a tube with iodine monochloride was inserted into the vapor phase above the reactants. The resulting iodolactone **26** was isolated in a modest yield (Table 3, entry 4). This reaction is already known to be a low yielding reaction [15]. One of the reasons for a low yield is that Diels-Alder reaction of cyclopentadiene and acrylic acid gives a large amount of the *exo* isomer, which cannot cyclize. The same reaction with dimethyl fumarate (**27**) in place of acrylic acid gave the corresponding iodolactone **28** in 87% yield. Cyclopentadiene and either acrylic acid or dimethyl fumarate react at a relatively high rate [26]. As most Diels-Alder reactions are relatively slow, and most dienes are highly reactive towards halogens, it may be necessary to carry out sequential reactions.

Solvent-free PV-PTFE reaction design is simple and inexpensive. With this experimental set up, reactions involving iodine monochloride worked well and we were able to avoid problems associated with a solid product coating PTFE tape. Bromine needed to be measured only if the stoichiometry was important (4-bromophenol) and was desirable when formation of byproducts was possible (2,4-dibromophenol). Usually, the end point of a reaction can be determined by color of bromine vapors persisting and the tube with unused bromine can be stored for future use. Alternatively, the end point was determined by replacing the delivery tube with a stopper and weighing the reaction flask taking into account the weight of bromine vapors. This reaction design avoids use of solvents and generates little or no waste while providing the reaction products in high yield and in high purity.

## Supporting Information

File 1 Additional experimental details and ^1^H NMR spectra of the isolated dibromostilbenes (**10** and **12**).
