# Supplementary material for: Solvent-free phase-vanishing reactions with PTFE (Teflon®) as a phase screen
Source: Beilstein J Org Chem. 2009 Dec 9;5:75. doi: 10.3762/bjoc.5.75 (PMC2839913; doi:10.3762/bjoc.5.75)
Supplement: File 1 — Additional experimental details and 1H NMR spectra of the isolated dibromostilbenes (10 and 12). [file Beilstein_J_Org_Chem-05-75-s001.pdf]

Supporting Information

Solvent-free phase-vanishing reactions with PTFE (Teflon®) as a phase screen

Kevin Pels and Veljko Dragojlovic*

Address: Wilkes Honors College of Florida Atlantic University, 5353 Parkside Drive, Jupiter, FL 33458, USA

Email: Veljko Dragojlovic - *vdragojl@fau.edu*

*Corresponding author

Table of contents

(1) Phase-vanishing PTFE (PV-PTFE) reaction setup 2
(2) General experimental methods 2
(3) Additional experimental details 2
(4) Experimental procedures 5
(5) NMR spectra of dibromostilbenes 7
(6) References 8

(1) Phase-vanishing PTFE (PV-PTFE) reaction setup

In a phase-vanishing PTFE (PV-PTFE) reaction setup we described in an earlier publication [1], the substrate in a solvent was placed in a reaction vessel, such as a vial, a test tube or a flask. The reagent was placed in a delivery vessel, such as a glass tube and sealed on both ends with PTFE tape. This tube was then inserted into the reaction vessel so that both reactants are in contact with the PTFE phase screen (Figure 1).

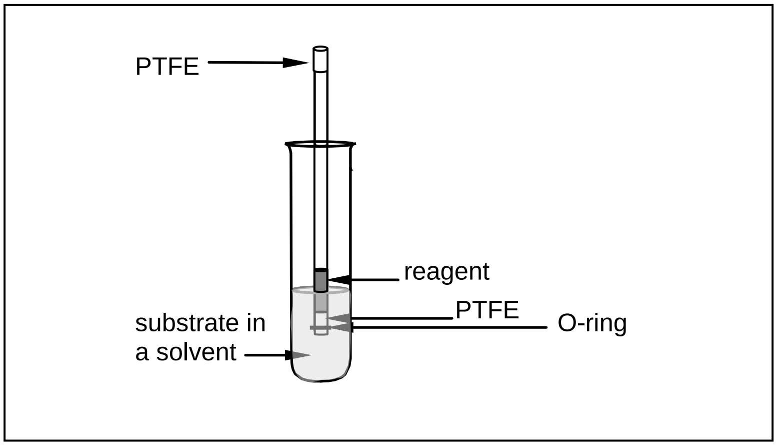

Figure 1: Phase-vanishing reaction with PTFE phase screen.

(2) General experimental methods

PTFE tape was commercially available Teflon® tape (High Density PTFE tape, Mil. Spec. T-27730A) made by Taega Technologies purchased from Fisher Scientific (cat. No. 14-610-120). If more than one layer of PTFE tape was used, the tape strips were applied perpendicular to each other in order to create a "mesh" of tape. It is important to secure PTFE tape either by means of an O-ring or additional PTFE tape wrapped along the walls of the delivery tube [1].

Acrylic acid, bromine, 4-bromophenol, cyclohexene, 2,4-dibromophenol, 2,3-dimethyl-2-butene, 3,3-dimethyl-1-butene, dicyclopentadiene, dimethyl fumarate, ethyl acetate, hexanes, iodine monochloride, 1-octene, 4-pentenoic acid, phenol, *trans*-stilbene and 2,4,6-tribromophenol were purchased from Acros Organics. 3-Butenoic acid and *cis*-stilbene were purchased from Aldrich. All of the reagents were used as supplied and without further purification. Cyclopentadiene is prepared by cracking dicyclopentadiene at 42 °C and used immediately.

Separations of the reaction products were carried out by preparative radial thin layer chromatography (Harrison Chromatotron). GC-MS analyses were performed by means of an Agilent 6890N Gas Chromatograph equipped with an HP-5MS 30 m × 0.25 mm column and an Agilent 5973N MSD. ^{1}H NMR spectra were recorded on a 400 MHz spectrometer in $CDCl_3$ solutions.

All of the isolated products were known compounds and gave satisfactory GC-MS and ^{1}H NMR data. Compounds **2, 14, 15** and **16** are commercially available and were identified by comparison with samples purchased from Aldrich. ^{1}H NMR spectra of the compounds (**4** [2], **6** [3], **8** [3], **10** [4], **12** [4], **18** [5], **19** [1], **20** [6], **22** [1], **23** [1], **26** [7], and **28** [8] have been reported in the literature.

(3) Additional experimental details

Use of a gas outlet. We used the apparatus shown below (Figure 2) for the reaction that proceeded with an evolution of a gas, or when there was a possibility for a drop in pressure.

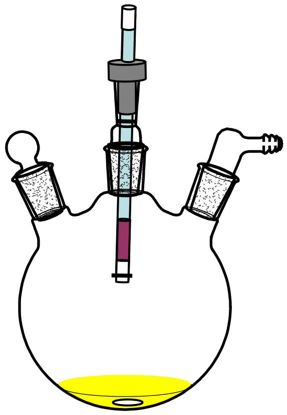

Figure 2: An alternative reaction apparatus.

Storage of filled delivery tubes. Filled delivery tubes can be stored for several days when inserted in a closed flask (Figure 3), provided that there are no leaks.

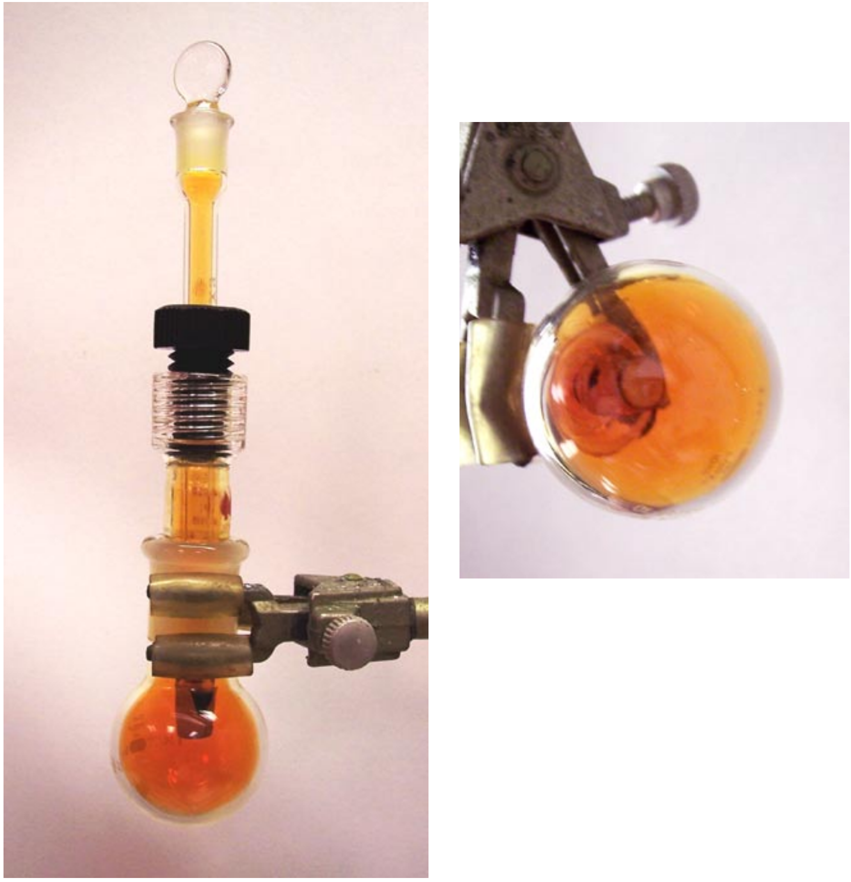

Figure 3: Storage of a bromine filled delivery tube.

To prevent loss of the reagent the top of the delivery tube should be closed. As, in the course of the reaction, the reagent is drawn into the reaction flask and consumed often resulting in a reduced pressure in the flask, a single layer of PTFE at the top of the tube is usually enough to prevent escape of the reagent such as bromine. However, as an added precaution, whenever tube design allowed it we placed either a

glass stopper (Figure 3) or a rubber septum to its top. In the course of a slow reaction, or if the filled reagent tube is to be stored, a stopper must be used.

Reactions on highly volatile substrates. In a reaction with highly reactive volatile substrates, the reaction occurred on PTFE surface and PTFE was coated with a solid product (Figure 4). Solid product dissolved some bromine, which often resulted in formation of byproducts. To avoid this, the reaction should be carried out at a low temperature and two layers of PTFE tape should be used.

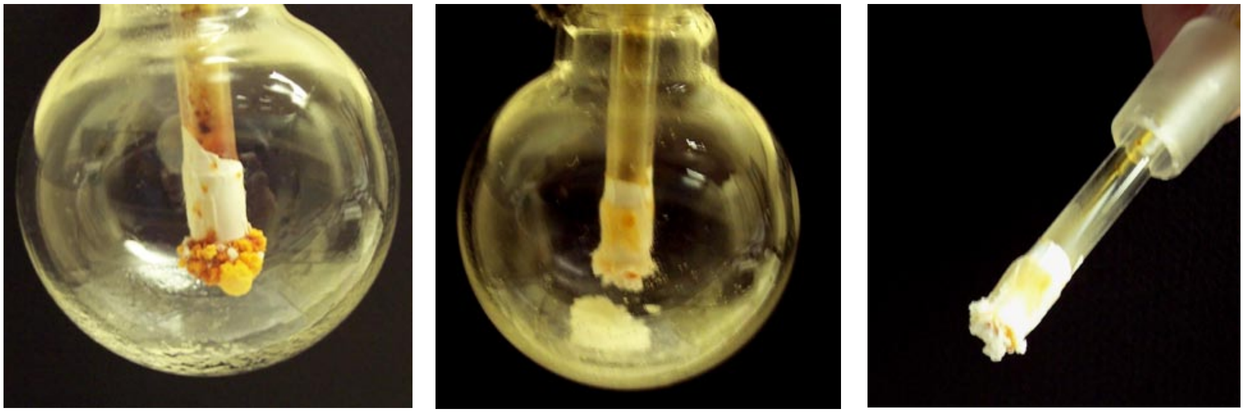

Figure 4: In a solvent-free PV-PTFE reaction involving highly reactive volatile substrates PTFE tape was coated with a solid product.

Reactions on liquid substrate that produces a solid product. When reaction on a liquid substrate produces a solid product adequate stirring is essential for good results. It is the best to employ a relatively large stirring bar and stir the mixture at a moderate rate, without splattering. In the absence of stirring, the solid product may form clumps that contain a large amount of starting material.

Additional safety considerations. If a solvent-free PV-PTFE reaction on highly reactive volatile substrates is carried out at a room temperature and the flask is allowed to be filled with the reagent vapors (e.g. by waiting for some time before inserting the reagent tube), there will be a sharp drop in pressure as in the course of a reaction the vapors are consumed to produce a non-volatile product. The difference in pressures inside the delivery tube (due to a column of the liquid reagent) and outside (reagent vapors condensed into a solid product) may cause the PTFE tape to slip off the tube and all of the reagent to be delivered into the flask. To avoid this, one should secure PTFE tape with an O-ring or additional PTFE wrap on the side [1]. In fact, we experienced slippage of the tape only in the reactions in solvent[1] and never in vapor-phase reactions described here. However, once cannot rule out that possibility. On a few occasions, when a reduced pressure was formed in the reaction flask, the reactant diffused in droplets through the PTFE screen. Usually one could observe formation of a drop at the end of the tube. The drop then slowly evaporated. When work up tube containing aqueous thiosulfate solution was used, on one occasion a drop of it fell into the flask. Thus, there is a possibility that the difference in pressure may cause the reagent to pass through the tape in droplets and be delivered too fast.
Possible solutions include use of two, or more, layers of PTFE tape, or carrying out the reaction at a low temperature (in an ice bath). We found that the best option was to incorporate a pressure equalizing gas outlet, as shown in Figure 2. If a gas was evolved, we used an appropriate gas trap (e.g. water or aqueous bicarbonate for HBr). If there was no evolution of a gas, a half-inflated balloon (filled with nitrogen) attached to the gas outlet was used as a pressure equalizing device.

(4) Experimental procedures

Addition of bromine to volatile reactive alkenes (Table 1, entries 1-4). A stirring bar and an alkene (2 mmol) were placed in a 10 mL round bottom flask. Flask was cooled in an ice bath and protected from light. PTFE-sealed bromine tube was inserted into the flask and was kept inside until the color of the bromine vapors persisted (5–10 min). At that point, bromine delivery tube was replaced with a PTFE-sealed tube filled with saturated aqueous solution of sodium thiosulfate. After the color of bromine vapors disappeared (~2–3 min) the product was collected from the flask.

Addition of bromine to *cis*-stilbene (Table 1, entry 5). A stirring bar and *cis*-stilbene (360 mg; 2 mmol) were placed in a 10 mL round bottom flask. PTFE-sealed bromine tube was inserted into the flask and was kept inside until the color of the bromine vapors persisted (15 min). At that point, bromine delivery tube was replaced with a PTFE-sealed tube filled with saturated aqueous solution of sodium thiosulfate. After the color of bromine vapors disappeared (~2-3 min) the product (0.653 g; 100%) was collected from the flask. According to ^{1}H NMR analysis it was composed of 91% D,L-dibromostilbene, 5% *trans*-stilbene and 4% *cis*-stilbene. ^{1}H NMR spectrum of the crude reaction product (in $CDCl_3$) is shown in Figure 5.

Addition of bromine to *trans*-stilbene (Table 1, entry 6). A stirring bar and solid *trans*-stilbene (360 mg; 2 mmol) were placed into a 10 mL round bottom flask. PTFE-sealed bromine tube was inserted and *trans*-stilbene powder was stirred vigorously. Periodically bromine delivery tube was replaced with a stopper and reaction flask was weighed. After the weight of the flask and its contents (*One should take the weight of bromine vapors into account!*) indicated that the addition was completed (60 min), bromine delivery tube was replaced with a PTFE-sealed tube filled with saturated aqueous solution of sodium thiosulfate. After the color of bromine vapors disappeared (~2–3 min) the product (0.667 g; 98%) was collected from the flask. According to ^{1}H NMR analysis it was composed of 96:4 mixture of *meso*-dibromostilbene and D,L-dibromostilbene. ^{1}H NMR spectrum of the crude reaction product (in acetone-d_6) is shown in Figure 6.

Bromination of phenol (Table 2, entries 1–5). A stirring bar and phenol (0.188 g, 2 mmol) were placed into a 10 mL round bottom flask. A bromine delivery tube was placed in the flask. The reaction was either carried out for the time stated, or a measured amount of bromine was used. Brominated phenol was collected from the flask.

Bromination of phenol (Table 2, entry 6). A stirring bar, water (0.5 mL) and phenol (0.188 g, 2 mmol) were placed into a 10 mL round bottom flask. A bromine delivery tube was placed in the flask. Bromine was consumed after 60 min. 2,4,6-Tribromophenol was collected by vacuum filtration and rinsed with water.

5-(Bromomethyl)dihydrofuran-2(3*H*)-one (**18**) **and 4,5-dibromopentanoic acid** (**19**) (**Table 3, entry 1**). A stirring bar and 0.200 g (2.0 mmol) of 4-pentenoic acid were placed into a 10 mL round bottom flask. A PTFE-sealed tube filled with 0.11 mL of bromine was inserted into the flask. Reaction was completed within 15 min. The product was dissolved in ethyl acetate and chromatographed. Chromatography (Harrison Chromatotron, eluting with 3:1 hexanes/ethyl acetate) afforded 0.201 g (56%) of 5-(bromomethyl)dihydrofuran-2(3*H*)-one (**18**) and 0.146 g (28%) of 4,5-dibromopentanoic acid (**19**).

5-(Iodomethyl)dihydrofuran-2(3*H*)-one (**20**) (**Table 3, entry 2**). A stirring bar and 0.200 g (2.0 mmol) of 4-pentenoic acid were placed into a 10 mL round bottom flask. A PTFE-sealed tube filled with 0.13 mL of iodine monochloride was inserted into the flask. Reaction was completed within 60 min. The product was dissolved in ethyl acetate and chromatographed. Chromatography (Harrison Chromatotron, eluting with 3:1 hexanes/ethyl acetate) afforded 0.407 g (90%) of 5-(iodomethyl)dihydrofuran-2(3*H*)-one (**20**).

4-Bromodihydrofuran-2(3*H*)-one (**22**) **and 3,4-dibromobutanoic acid** (**23**) (**Table 3, entry 3**). A stirring bar and 0.175 g (2.0 mmol) of 3-butenoic acid were placed into a 10 mL round bottom flask. A PTFE-sealed tube filled with 0.11 mL of bromine was inserted into the flask. Reaction was completed within 10 min. The product was dissolved in ethyl acetate and chromatographed. Chromatography (Harrison Chromatotron, eluting with 3:1 hexanes/ethyl acetate) afforded 0.069 g (21%) of 4-bromodihydrofuran-2(3*H*)-one (**22**) and 0.330 g (67%) of 3,4-dibromobutanoic acid (**23**).

Iodolactone 26 (**Table 3, entry 4**). A stirring bar, 0.25 mL (3.0 mmol) of cyclopentadiene and 0.22 g (3.0 mmol) of acrylic acid were placed into a 25 mL round bottom flask. A PTFE-sealed tube with 0.20 mL (4.0 mmol) iodine monochloride, was inserted into the flask. The mixture was stirred for 1 h. The product was dissolved in ethyl acetate and chromatographed. Chromatography (Harrison Chromatotron, eluting with 3:1 hexanes/ethyl acetate) afforded 0.297 g (38%) of iodolactone **26**.

Iodolactone ester 28 (**Table 3, entry 5**). A stirring bar, 0.42 mL (5.0 mmol) of cyclopentadiene and 0.72 g (5.0 mmol) of dimethyl fumarate were placed into a 25 mL round bottom flask. A PTFE-sealed tube with 0.30 mL (6.0 mmol) iodine monochloride, was inserted into the flask. The mixture was stirred for 6 h. The product was dissolved in ethyl acetate, treated with aqueous sodium thiosulfate and filtered through a short column of silica gel (2.5 cm x 5 cm, eluting with 1:1 hexanes/ethyl acetate) to give 1.401 g (87%) of iodolactone ester **28**.

(5) NMR spectra of dibromostilbenes

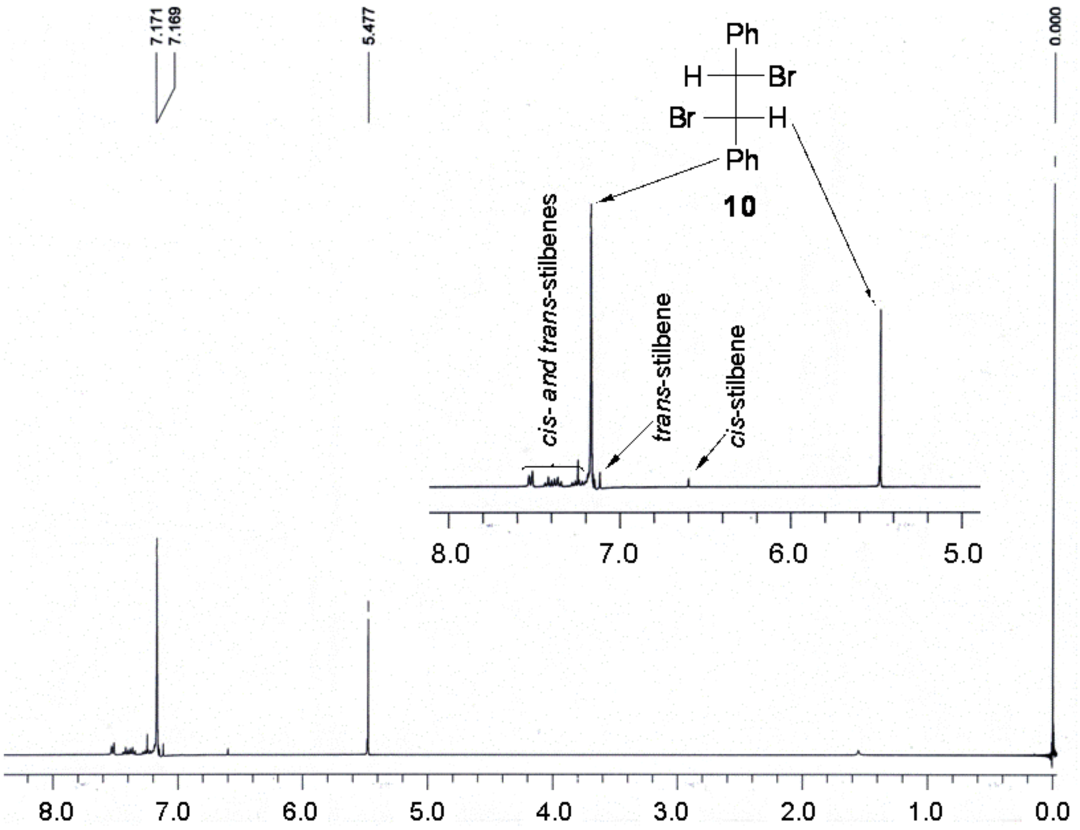

Figure 5: ^{1}H NMR spectrum of crude D,L-dibromostilbene (**10**) (in $CDCl_3$) obtained in the course of a solvent-free PV-PTFE reaction.

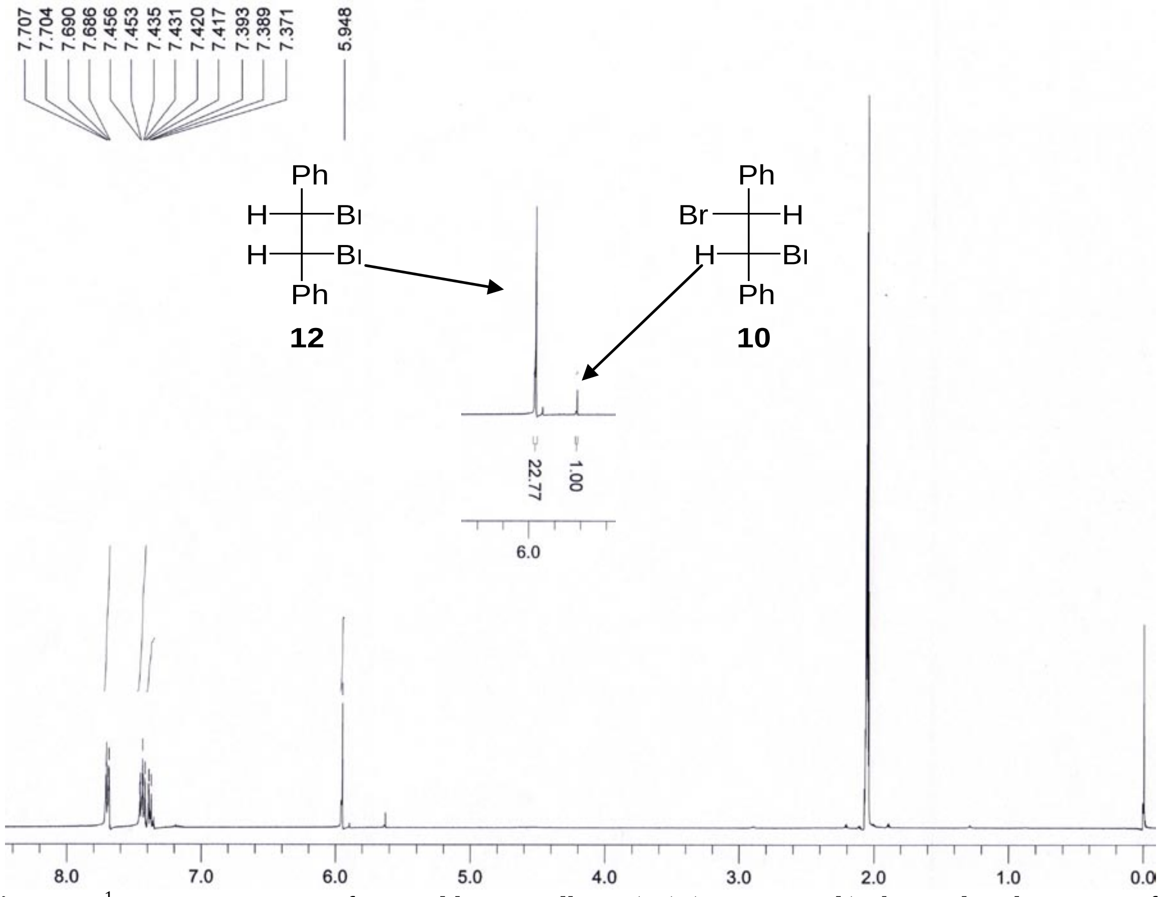

Figure 6: ^{1}H NMR spectrum of *meso*-dibromostilbene (**12**) (in acetone-d_6) obtained in the course of a solvent-free PV-PTFE reaction.

(6) References

1. Van Zee, N. J.; Dragojlovic, V. *Org. Lett.* **2009,** *11,* 3190–3193. doi:10.1021/ol901450h
2. Adimurthy, S.; Ghosh, S.; Patoliya, P. U.; Ramachandraiah, G.; Agrawal, M.; Gandhi, M. R.; Upadhyay, S. C.; Ghosh, P. K.; Ranu, B. C. *Green Chem.* **2008,** *10,* 232–237. doi:10.1039/b713829f
3. Integrated Spectral Database System of Organic Compounds (web site maintained by the National Institute of Advanced Industrial Science and Technology (AIST), Japan). http://riodb01.ibase.aist.go.jp/sdbs/cgi-bin/cre_index.cgi?lang=eng (accessed on Aug 3, 2009).
4. Ma, K.; Li, S.; Weiss, R. G. *Org. Lett.* **2008,** *10,* 4155–4158. doi:10.1021/ol801327n
5. Abe, M.; You, Y.; Detty, M. R. *Organometallics* **2002,** *21,* 4546–4551. doi:10.1021/om020511p
6. Curran, D. P.; Tamine, J. *J. Org. Chem.* **1991,** *56,* 2746–2750. doi:10.1021/jo00008a032
7. Moriarty, R. M.; Gopal, H.; Walsh, H. G.; Ramey, K. C.; Lini, D. C. *Tetrahedron Lett.* **1966,** 4555–4560. doi:10.1016/S0040-4039(00)70077-3
8. Kishikawa, K.; Horie, K.; Yamamoto, M.; Kohmoto, S.; Yamada, K. *Chem. Lett.* **1990,** 1009–1010. doi:10.1246/cl.1990.1009
